# The use of social media to supplement resident medical education – the SMART-ME initiative

**DOI:** 10.3402/meo.v21.29332

**Published:** 2016-01-08

**Authors:** Panagis Galiatsatos, Fernanda Porto-Carreiro, Jennifer Hayashi, Sammy Zakaria, Colleen Christmas

**Affiliations:** Department of Internal Medicine, Johns Hopkins University School of Medicine, Baltimore, MD, USA

**Keywords:** social media, Twitter, medical education

## Abstract

**Background:**

Residents work at variable times and are often unable to attend all scheduled educational sessions. Therefore, new asynchronistic approaches to learning are essential in ensuring exposure to a comprehensive education. Social media tools may be especially useful, because they are accessed at times convenient for the learner.

**Objective:**

Assess if the use of Twitter for medical education impacts the attitude and behavior of residents toward using social media for medical education.

**Design:**

Preintervention and postintervention surveys. Internal medicine resident physicians were surveyed before the launch of a residency-specific Twitter webpage on August 1, 2013, and again 135 days later, to determine their use of the Twitter application and web page, as well as other social media for medical education.

**Participants:**

Residents at an internal medicine urban academic training program.

**Main Measures:**

All residents within our training program were administered web-based surveys. The surveys assessed resident views and their frequency of use of social media for medical education purposes, and consisted of 10 Likert scale questions. Each answer consisted of a datapoint on a 1–5 scale (1=not useful, 3=useful, 5=very useful). The final survey question was open-ended and asked for general comments.

**Key Results:**

Thirty-five of 50 residents (70%) completed the presurvey and 40 (80%) participated in the postsurvey. At baseline, 34 out of 35 residents used social media and nine specifically used Twitter. Twenty-seven (77%) used social media for medical education; however, only three used Twitter for educational purposes. After the establishment of the Twitter page, the percentage of residents using social media for educational purposes increased (34 of 40 residents, 85%), and 22 used Twitter for this purpose (*p<*0.001 for the change). The percentage of residents using the application at least once a week also increased from 11.4 to 60.0% (*p<*0.001). Almost all residents (38 of 40) felt that social media could be useful as a medical education tool, which slightly increased from 30 out of 35 in the preintervention survey (*p=*0.01).

**Conclusion:**

Residents believe social media could be used for medical education. After we launched a Twitter page for medical education, there was a significant increase in the use and frequency of Twitter for resident medical education over the ensuing 6 months. Further research should be performed to see if social media can impact overall medical knowledge and patient care, and whether longer term use is maintained.

Online applications and websites where users contribute, retrieve, and explore content generated by fellow users are popular means to share and communicate information. As an example, Facebook, the most utilized social media application, is used by more than 1.19 billion users every month and has 727 million daily users (1). Within the United States, social media sites are extremely popular, with an estimated 18 to 39 million people checking social media sites multiple times per day (2). Given this popularity, many health care professionals have utilized social media as a means to promote medical information and education, which is associated with a number of benefits as well as concerns (3–10).

Social media may be especially useful for enhancing medical education. Restrictions on resident work hours may limit their ability to participate in in-person conferences and educational sessions. Social media can be accessed geographically and temporally in asynchronous manners, allows for easily searchable and stored content, and encourages interactivity (8) – and thus may offer certain advantages over more traditional educational formats. Furthermore, the majority of health professional students prefer online media as their primary source of information (11) and millennial learners have come to expect it as part of their education (12). To address these concerns and explore the use of social media at our urban academic internal medicine residency training program, we started using audio recordings, or ‘podcasts’, to supplement morning reports, because on any given day up to one-third of our residents are unable to attend in person. Specifically, we created weekly podcasts with a running time of 7–10 min that highlighted the most important learning points. These podcasts were well received, with residents appreciating their effectiveness as an educational supplement (13).

Buoyed by the success of the podcasts, residents sought additional tools to enhance their medical education. We then chose Twitter, an online social networking service, which allows the exchange of information in ‘tweets’, messages shorter than 140 characters (14), potentially providing pithy and memorable ‘pearls’ to reinforce conference teachings. In contrast to Facebook and other personal sites, Twitter is designed to facilitate discussions and share ideas (15). Reports describe that some physicians use Twitter to promote health to the general public (16). We sought to study the impact of Twitter as an educational supplement on resident attitudes and behavior regarding the use of social media for medical education.

## Methods

**Fig. 1:**
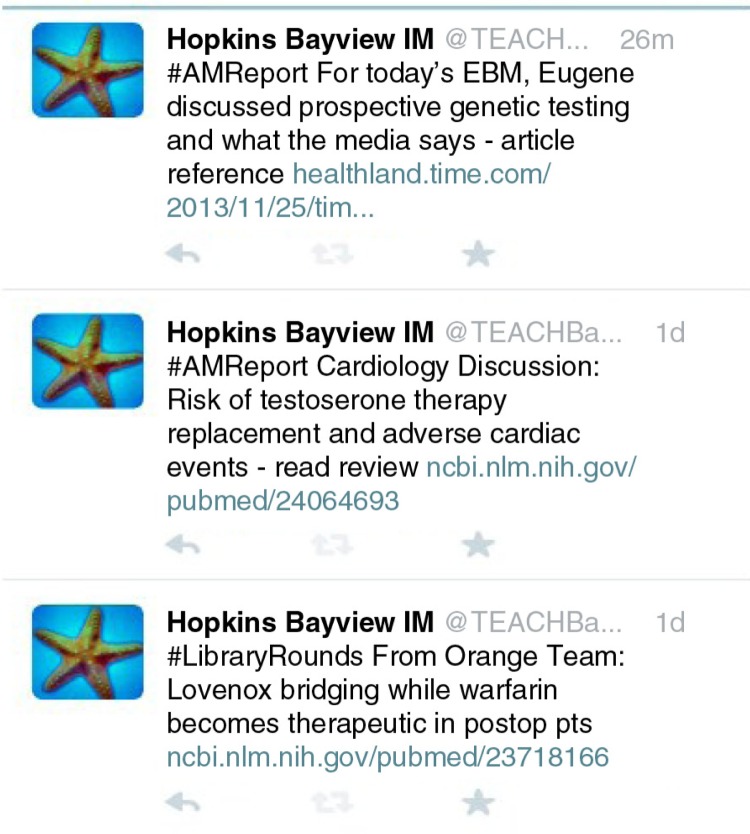
Snapshot of @TEACHBayview Twitter page.

We launched the Social Media and Resident Teaching for Medical Education initiative at Johns Hopkins Bayview Medical Center, a tertiary care academic institution located in Baltimore, Maryland. On August 1, 2013, we introduced a Twitter page, titled ‘Hopkins Bayview IM @TEACHBayview’. This page listed a series of Twitter messages (called ‘tweets’). Each ‘tweet’ can contain up to 140 characters and can include photos, videos, and links which, for instance, can reference an online article. In addition, the page included ‘hashtags’, which are keywords preceded by the # character (17). Each hashtag simplifies searching and categorizing topics. By clicking on a specific hashtag, all of the tweets marked by the keyword are listed, even if tweeted by different Twitter providers. In addition, the hashtag, which can occur anywhere in a tweeted message, can help messages become popular, known as ‘trending’ (17). Figure 1 provides a screen shot detailing tweets with three hashtags.

The material posted in a Twitter message serves as a summary of one educational lesson, and is categorized into one of six types (as represented by a hashtag). The six hashtags used by @TEACHBayview are detailed in Table 1 and are #AMreport, #ChiefsRounds, #LibraryRounds, #NoonConference, #DocOfTheDayPearl, and #AlumniNews. During the intervention time period, a total of 200 tweets were sent by @TEACHBayview over the 135-day period: 107 under #AMreport, 32 under #LibraryRounds, 20 under #NoonConference, 18 under #DocOfTheDayPearl, 12 under #ChiefsRounds and 1 under #AlumniNews. The daily tweet rate was one to two per day.

**Table 1:** Hashtags used by @TEACHBayview

#AMReport – Highlights relevant articles or teaching pearls from morning report.
#NoonConference – Provides access to articles cited by the noon conference faculty presenter.
#ChiefsRounds – Highlights learning points from teaching rounds led by senior faculty. Prior to Twitter, only one ward team at a time were aware of the educational discussions.
#DocOfTheDayPearl – Disseminates primary care-related teaching points identified by a senior resident who is rotating as the urgent-care outpatient provider for the resident clinic.
#LibraryRounds – Identifies evidence-based literature topics researched by an informationist who rounds daily with one Medicine Wards team.
#AlumniNews – Highlights resident alumni publications.

Two weeks prior to the Twitter page launch, we invited all 50 internal medicine residents to complete a voluntary baseline survey, which queried the quantity and type of social media used by residents for medical education purposes. Specifically, the survey used Likert scales to quantify resident attitudes toward social media use for medical education (1=not useful, 3=useful, 5=very useful), and addressed the frequency of social media use (including Twitter) for medical education (1=rarely, less than once a month to 5=multiple times a day). Finally, free response questions were asked about resident attitudes toward the ‘Hopkins Bayview IM @TEACHBayview’ Twitter page.

To assess the impact of the Twitter page on resident behavior and attitudes toward social media use for medical education we repeated the survey 135 days after launch of the Twitter page. Furthermore, we also reviewed the use of other types of social media by residents before and after the implementation of our Twitter page. Pre- and post-survey results were analyzed using Student *t*-tests to determine statistical significance in changes. Where appropriate, scores are shown as mean (±standard deviation). Qualitative responses are shown in a separate table.

## Results

Of the 50 residents, 35 residents participated in the presurvey and 40 in the postsurvey. Residents extensively used social media (34 of 35 in the presurvey (97.1%) and 37 of 40 in the postsurvey (92.5%) (*p=*0.19)). In the presurvey, most used Facebook (31 residents), YouTube (24 residents), and podcasts (17 residents). In contrast, Twitter was used by only nine residents. After the launch of the Hopkins Bayview Twitter page, Facebook and YouTube were still the most frequently used social media (34 and 28, respectively, *p=*0.71). However, Twitter use increased from 9 residents to 23 (*p<*0.01) and became the third most used social media application.

**Fig. 2:**
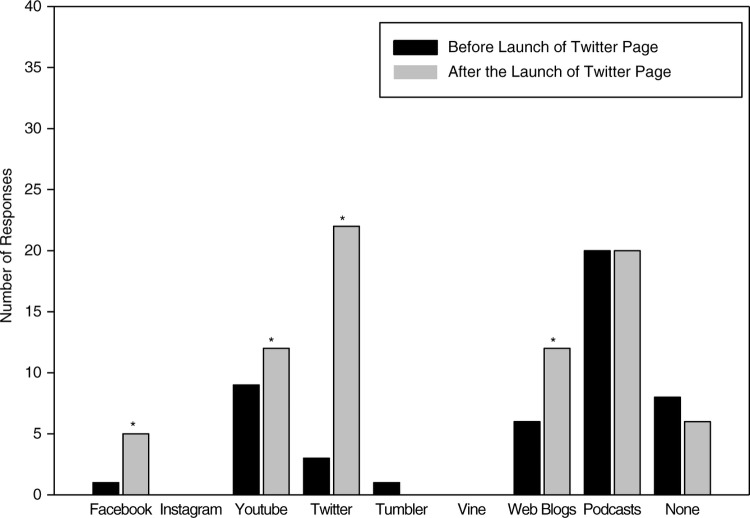
Social media use by the residents for medical education. * indicates *p*<0.05.

Specific to medicine, 27 of the 35 residents in the presurvey used social media tools to enhance their education (Fig. 2), with podcasts (20 residents), YouTube (9 residents), and weblogs (6 residents) being the top three applications. However, usage was infrequent, with 13 residents using social media less than once a month followed by 19 who use it up to twice a week, with only three residents using it daily. After the launch of our Twitter page, 34 of the 40 residents used social media (77% presurvey to 85% postsurvey, *p=*0.28), which was not statistically different. For frequency of use, postintervention surveys revealed that 21 residents used social media up to twice a week and 10 residents used social media daily. Nine residents continued to rarely use (less than once a month) social media for medical education. As with use of social media, the frequency of using social media for medical education at least once a week was not statistically significant: 22 of 35 residents versus 31 of 40 (62.8% vs. 77.5%, respectively, *p=*0.08).

After our Twitter page was launched, postsurveys showed it was the most popular source for medical education, with 22 of the 40 residents using it for that purpose, compared to 3 of the 35 residents prior to the intervention (*p<*0.001). In addition, there were also statistically significant increases in using other social media for medical education: Facebook (1 to 5, *p=*0.002), YouTube (9 to 12, *p=*0.04), and weblogs (6 to 12, *p=*0.003). However, podcast use did not change (20 residents before and after the surveys). Also, six residents continued to not use social media for medical education purposes (decreased from eight in the presurvey, *p=*0.09).

**Fig. 3:**
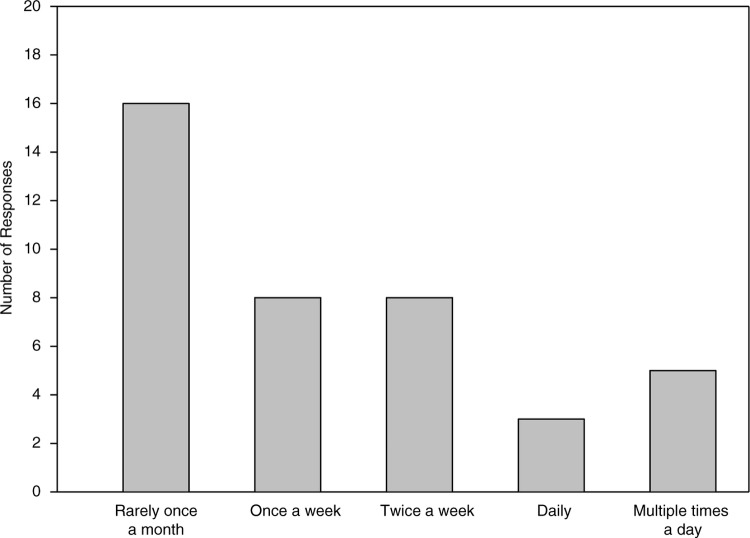
Frequency of Twitter use by residents for medical education.

The frequency of Twitter use for medical education also increased. Preintervention, four residents accessed the application weekly, with one accessing Twitter feeds through other social media platforms (Fig. 2). Postintervention, more residents accessed Twitter at least weekly, from 11.4 to 60.0% (*p<*0.001) (Fig. 3).

Resident views also changed, with more favorable views on social media for medical education (3.56±1.08 vs. 3.61±1.18 (*p=*0.01)). In open-ended questions, resident views on Twitter are listed in Table 2, and are mostly favorable.

**Table 2:** Open ended resident responses regarding likes and dislikes about the Twitter feed

Positive remarks	Negative remarks; areas for improvement
‘I have just started using Twitter – was resistant, but have found it a great tool. Keeps me updated’. ‘No changes’ ‘Love it though took some time to catch on’ ‘I like that it's an easy way to read a pertinent article’. ‘Great way to update myself on things discussed at morning report and noon conference when I'm not able to go! And also a resource for article links and other useful additive material’. ‘It has freed my email from clusters ‘read this paper’ or ‘update from morning report’ – it allows me to learn at my own time’. ‘I like being able to easily find the articles and things we were discussing’. ‘Easy access to papers discussed during morning report/lunch talks’. ‘I like the links to useful info and articles’. ‘Bite size information’.	‘The reason that I don't go onto Twitter is that I feel like it's just another time distraction like Facebook. Even though it's for educational purposes, I know that I'd waste time looking at other Twitter feeds’. ‘Love it but would be great to have chats around images or cases!’ ‘Maybe ways teams can collaborate for patient care’. ‘I do not use twitter often enough for this to be useful’. ‘I was using my twitter, and was a good learning portal. But then my account got hacked, which deterred me from continual use’. ‘I also wish a mystery case was featured periodically’. ‘I wish there was more stuff posted more often!’ ‘I still have not joined – no comment’.

## Discussion

Although many residents thought that social media would be useful for medical education, more residents began to frequently use Twitter and other social media after launch of the Twitter page. Resident views toward social media and medical education also changed, revealing a stronger desire for using social media to supplement their learning. Multiple free-response comments revealed Twitter's value for real-time information and relevant literature article links.

Although there are no cited examples specifically using Twitter for internal medicine resident education, others have successfully used Twitter as a medical educational adjunct, which can lead to greater dissemination of learning points. As an example, a Twitter page designed to summarize teaching in a fourth-year medical school ultrasound training exercise (18), ultimately was followed by a large group (37%) of interns, residents, and attendings, none of whom actually took the course. Similar to our followers, the majority of followers had never previously used Twitter (55.6%), but found Twitter to be user-friendly (88.9%) and useful (81.5%). As a result, many followers (59.2%) wanted to follow more educational feeds via Twitter. In the graduate medical education sphere, Twitter has been successfully used to communicate with various stakeholders (e.g., residents, attendings) in academic radiology departments (19). Twitter may be especially useful for this image-based specialty, because each tweet can contain images or other multimedia. We had similar success with sharing radiographic images, as well as other pictures (e.g., electrocardiograms, flow-volume wave forms). Furthermore, the authors found that half of the content that these departments tweeted were promotional – awards, research publications, upcoming events – and often led to ‘retweets’. We used one hashtag for promotional purposes, #AlumniNews, which, too, often led to trending. Thus, other medical specialties, in this case radiology, have found similar success using Twitter for educational purposes, as well as promotional.

There are concerns with the use of social media outlets for medical education, including Twitter (20–23). First, Health Insurance Portability and Accountability Act (HIPAA) standards need to be always followed. For example, our #AMreport tweets come from learning points obtained from morning reports, where protected patient information is discussed for educational purposes. In each tweet, the content of the tweet must be stripped of all protected identifying information. Second, the use of social media was primarily designed for the public sharing of non-medical information. Much of this information is of a personal nature, and does not conform to professional medical standards. When using social media for educational purposes, we may encounter challenges in shifting the mindset behind each tweet to emphasize medical professionalism when sharing learning points. Third, social media use can lead to distribution of copyrighted material, so each tweet needs to be carefully reviewed in order to avoid inappropriately distributing anything protected under copyright laws. Finally, the volume of information exchange through social media sites is extraordinary. It can be hard to effectively use social media to convey medical teaching points among a number of other tweets devoted to other topics.

We should note that the surveys were voluntary and thus had variable completion rates (35 in presurvey and 40 in postsurvey). Also, we did not obtain demographic data, which was also the case in other publications studying social media (8, 18, 19). Finally, we chose to study a less popular social media tool; although Twitter offers great advantages, residents had to familiarize themselves with this application without formal training. Despite these limitations, we are encouraged by our survey results and conclusions, and believe that social media has an important role in medical education.

In conclusion, using Twitter to convey and discuss medical concepts has a significantly beneficial impact on internal medicine residents. Residents are favorably inclined to use the application and welcome greater use of social media to supplement their medical education. However, it is unclear whether social media, or Twitter itself, truly impacts resident learning or promotes better patient care. Future studies should address these issues.

## Conflict of interest and funding

The authors have not received any funding or benefits from industry or elsewhere to conduct this study.
